# Modeling of Dysferlinopathy (LGMDR2) Progression: A Longitudinal Fat Fraction Analysis

**DOI:** 10.1212/NXG.0000000000200283

**Published:** 2025-07-29

**Authors:** Carla Florencia Bolano-Diaz, Harmen Reyngoudt, Ian J. Wilson, Meredith K. James, Fiona Elizabeth Smith, Ericky Caldas de Almeida Araujo, Heather Gordish-Dressman, Heather Hilsden, Laura E. Rufibach, Dorothy Wallace, Louise Ward, Roberto Stramare, Alessandro Rampado, Mark Smith, Jean-Marc Boisserie, Julien Le Louer, Sheryl Foster, Anthony Peduto, Noriko Sato, Takeshi Tamaru, Anne Marie Sawyer, John W. Day, Kristi J. Jones, Maggie Christine Walter, Tanya Stojkovic, Madoka Mori-Yoshimura, Jerry R. Mendell, Elena Pegoraro, Volker Straub, Andrew M. Blamire, Pierre Carlier, Jordi Diaz-Manera, for the Jain COS Consortium, Marni Jacobs

**Affiliations:** 1 The John Walton Muscular Dystrophy Research Centre, Translational and Clinical Research Institute, Newcastle University and Newcastle Hospitals NHS Foundation Trust, Central Parkway, Newcastle Upon Tyne, United Kingdom;; 2 NMR Laboratory, Neuromuscular Investigation Centre, Institute of Myology, Paris, France;; 3 Newcastle Magnetic Resonance Centre, Translational and Clinical Research Institute, Newcastle University, United Kingdom;; 4 Wolfson ACTIVE Laboratory, Institute of Sport, Manchester Metropolitan University, United Kingdom;; 5 Center for Translational Science, Division of Biostatistics and Study Methodology, Children's National Health System, Washington, DC;; 6 Pediatrics, Epidemiology and Biostatistics, George Washington University, Washington, DC;; 7 The Jain Foundation, Seattle, WA;; 8 Radiology Unit, Department of Medicine, University of Padova, Italy;; 9 Department of Radiology, Nationwide Children's Hospital, Columbus, OH;; 10 Department of Radiology, Westmead Hospital; Faculty of Health Sciences, The University of Sydney, Australia;; 11 Department of Radiology, National Center Hospital, National Center of Neurology and Psychiatry, Tokyo, Japan;; 12 Lucas Centre for Imaging, Stanford University School of Medicine, CA;; 13 Department of Neurology and Neurological Sciences, Stanford University School of Medicine, CA;; 14 Sydney Children's Hospital Network, Sydney, Austrialia;; 15 University of Sydney, Australia;; 16 Friedrich Baur Institute at the Department of Neurology, LMU University Hospital, LMU Munich;; 17 Institut de Myologie, AP-HP, Hôpital Pitié-Salpêtrière, Centre de référence des maladies neuromusculaires Nord/Est/Ile de France, Paris, France;; 18 Department of Neurology, National Center Hospital, National Center of Neurology and Psychiatry Tokyo, Japan;; 19 Nationwide Children's Hospital, Columbus, OH;; 20 Department of Neuroscience, University of Padova, Italy; and; 21 St Luc University Hospital, Erasme University Hospital, Brussels and University of Liege, Belgium.

## Abstract

**Background and Objectives:**

Limb-girdle muscular dystrophy R2 (LGMDR2) is characterized by progressive muscle weakness usually leading to severe disability. The rate of progression and disease severity is variable among patients, although factors influencing this variability are not completely understood. The Dysferlinopathy Clinical Outcome Study is a natural history study that followed patients with LGMDR2 for 3 consecutive years using functional outcome measures and skeletal muscle MRI.

The aim of our study was to develop statistical models able to describe fat fraction (FF) progression of the lower limbs in patients with LGMDR2 using clinical and radiologic variables to better understand which factors influence disease progression and improve the design of future clinical trials.

**Methods:**

We used linear-mixed modeling to analyze changes in FF over time according to patients' age. We calculated the average FF trajectory for each muscle of the lower limbs. We built 2 multivariate models for each segment adding other clinical factors and using likelihood ratio test and residuals' analysis to determine whether they better fitted observed FF values.

**Results:**

Muscles that participated in the same joint movement progressed similarly over time. FF was expected to be higher the older patients were and the earlier the age at symptom onset. Women had absolute FF values 8.8% higher than men in the lower leg. No differences in FF trajectory were seen based on ethnic groups (White, Asian, Black, or Hispanic), genetic variants, or residual dysferlin expression. Although multivariate models showed a better global fit to the data, there was no improvement in representing individual patient variability.

**Discussion:**

In conclusion, this study provides a better understanding of skeletal muscle fat replacement progression in the lower limb muscles of patients with LGMDR2, highlighting the influence of age at symptom onset, sex, and baseline motor function, which should be considered in the design and analysis of clinical trials. Although complex models improved the overall data fit, they did not improve the accuracy in identifying changes at a patient level, underlying the need for further research and validation and the fact that other variables we have not measured are probably influencing progression.

## Introduction

Dysferlinopathy is a form of limb-girdle muscular dystrophy (LGMDR2) produced by pathogenic variants in the *DYSF* gene that encodes the protein dysferlin.^1^ It is inherited in an autosomal recessive manner, and its main symptom is muscle weakness. Dysferlin is located at the sarcolemma of the muscle fibers and at the T tubules, playing a key role in the repair process of the membrane after injury^2,3^ and in cell calcium handling.^4,5^ Its absence or malfunction leads to a defective sealing of the muscle fiber, triggering a cascade of molecular consequences eventually leading to muscle fiber death.^6^ Muscle fibers become progressively replaced by connective and fat tissue, which do not have any contractile properties, leading to irreversible muscle weakness and wasting. Although muscle atrophy and weakness inevitably progress over time, the rate and severity at which this happens is variable, even in patients with the same underlying pathogenic variants belonging to the same family.^7,8^

The Dysferlinopathy Clinical Outcome Study 1 (COS 1) is an international, natural history study, funded by the Jain Foundation, through which longitudinal clinical, genetic, and imaging data were collected in 15 sites across Spain, Germany, Italy, France, the United States, Japan, Australia, and the United Kingdom.^7^ Several conclusions were drawn from the data analysis regarding disease progression: Rate of disease progression did not differ according to the initial phenotype^9^; a steeper decline in function was seen during the first 10 years of disease^10^; younger age at onset was associated with worse function and faster disease progression^10^; Asian patients performed worse than White patients in timed tests across the disease course^10^; intense physical activity during teenage years, which can lead to higher rates of muscle injury, was associated not only with earlier symptom onset and earlier need for a wheelchair full-time^11^ but also with more severe muscle replacement by fat in MRI^12^; and higher baseline water T_2_ values were linked to greater functional decline during the next 3 years of progression.^13^ However, some conflicting results were more difficult to integrate, for example those regarding sex differences. Although women scored better in the North Star Assessment for Dysferlinopathy (NSAD) at baseline,^9^ they simultaneously showed higher fat content in several muscles measured through MRI^13,14^ Although current findings offer insight into certain aspects of disease progression, contradictions and persistent knowledge gaps highlight the complexity of LGMDR2 and the need for further research to fully understand its natural history.

MRI is increasingly being implemented as an outcome measure in clinical trials, both natural history and interventional studies, because it not only correlates well with clinical changes,^15-18^ but it can also overcome some of the limitations clinical assessments face, such as variability because of external uncontrollable factors (from the patient and evaluator), inability to measure loss of function in presymptomatic patients,^19,20^ and less sensitivity in detecting changes over a short period of time.^21^ A partial longitudinal analysis of COS 1 MRI data involving 54 patients from 2 sites and 12 healthy controls was published in 2022,^15^ assessing fat fraction (FF), contractile cross sectional area, muscle water T_2,_ and phosphorus MR spectroscopy in the lower limbs, providing broader multidimensional information on MRI findings of disease progression. To describe disease trajectories in a more accurate and thorough way, not only clinical changes should be considered but also changes in MRI-based variables. Moreover, with novel potential disease-modifying therapies being closer to the human trials stage, a clearer understanding of the natural history of muscular dystrophies and of how different outcome measures correlate and influence each other over time is essential.

Our main aim was to characterize natural disease severity and progression in patients with LGMDR2 by modeling quantitative MRI FF measurements of the skeletal muscles of the lower limbs obtained longitudinally, include clinical variables into the models to improve their fit to the FF data, and assess how these variables influence the muscle replacement by fat process over time.

## Methods

### Study Design and Participants

COS 1 collected clinical, genetic, histologic, biochemical, and imaging data of dysferlin-deficient patients over a 3-year period. Patients who provided written informed consent and had 2 pathogenic variants or 1 pathogenic variant plus absent dysferlin expression through immunoblot in either muscle or monocytes were included. It was initially approved on February 2, 2012, by the NRES Committee North East–Newcastle and North Tyneside (reference 211/NE/0360/R&D 5918), and being an international collaborative study, it was also approved by ethical review boards in each country (a more detailed description of the methods can be found in the first paper published from COS 1^7^).

MRI assessment included T_1_-weighted images of the whole body, and T_2_-weighted, 2-point or 3-point Dixon and MR spectroscopy of the lower limbs. These images were acquired at baseline and yearly after that.

### MRI Acquisition and Image Analysis

eTable 1 summarizes the MR Dixon acquisition parameters for all participating sites, and detailed description of the acquisition parameters and image analysis for the Newcastle and Paris sites can be found in the materials and methods section of the paper where these data was first published.^15^ Bilateral FF data were available for the following muscles: the vastus intermedius, vastus lateralis, and vastus medialis (anterior thigh compartment or femoral quadriceps); the adductor magnus (AM), gracilis, and sartorius (medial thigh compartment); the biceps femoris, semitendinosus, and semimembranosus (posterior thigh compartment or hamstrings); the tibialis anterior and extensor digitorum muscles (anterior lower leg compartment); the peroneals (lateral lower leg compartment); and the tibialis posterior (TP), soleus, gastrocnemius medialis, and gastrocnemius lateralis (posterior lower leg compartment). Using these values, we calculated the mean FF for the thigh and lower leg.

**Table 1:** Clinical and Imaging Characteristics at Each Visit

Variables (mean ± SD)	Baseline	Year 1	Year 2	Year 3
Age at visit (yo)	37.52 ± 11.37	38.66 ± 11.15	39.59 ± 11.16	39.99 ± 10.71
Disease duration (y)	15.98 ± 9.15	16.98 ± 8.52	18.24 ± 9.21	18.26 ± 8.17
Age at symptom onset (yo)	22.43 ± 8.60	NA	NA	NA
CK (U/L)	4,734.57 ± 4,016.78	4,401.33 ± 3,317.74	3,894.15 ± 2,800.00	3,652.83 ± 2,664.52
NSAD (X/54)	29.38 ± 15.11	28.43 ± 15.55	20.65 ± 15.84	18.79 ± 15.8
Thigh FF (%)	41.93 ± 23.34	45.98 ± 23.37	48.13 ± 22.78	49.01 ± 21.10
Anterior compartment				
VI FF (%)	43.26 ± 26.00	47.3 ± 26.19	49.45 ± 25.10	49.9 ± 22.90
VL FF (%)	41.69 ± 26.04	45.31 ± 25.73	48.84 ± 25.34	48.39 ± 23.14
VM FF (%)	41.19 ± 24.63	45.44 ± 25.22	48.32 ± 24.48	47.6 ± 22.37
Posterior compartment				
BF FF (%)	47.16 ± 26.59	50.03 ± 26.42	52.71 ± 25.95	55.51 ± 24.84
SM FF (%)	56.09 ± 26.07	60.28 ± 24.00	60.98 ± 24.82	61.7 ± 23.01
ST FF (%)	45.34 ± 27.44	50.03 ± 26.92	52.60 ± 26.09	54.73 ± 24.06
Medial compartment				
AM FF (%)	47.00 ± 26.06	52.00 ± 25.5	53.59 ± 25.44	55.77 ± 23.83
Gra FF (%)	26.82 ± 24.58	31.38 ± 25.46	31.93 ± 26.05	32.57 ± 25.72
Sar FF (%)	29.71 ± 25.07	33.21 ± 26.56	34.74 ± 27.28	34.91 ± 26.73
Lower leg FF (%)	41.12 ± 22.63	42.31 ± 21.09	46.73 ± 20.03	44.87 ± 19.30
Anterior compartment				
ED FF (%)	30.28 ± 24.99	31.61 ± 24.08	34.94 ± 24.49	33.88 ± 23.00
TA FF (%)	34.65 ± 25.32	35.46 ± 24.22	39.23 ± 23.54	38.44 ± 22.66
Posterior compartment				
TP FF (%)	32.98 ± 25.25	33.2 ± 23.72	37.11 ± 25.07	36.19 ± 24.28
GL FF (%)	44.01 ± 26.70	46.36 ± 24.97	52.63 ± 22.46	49.49 ± 22.25
Sol FF (%)	49.69 ± 25.64	50.42 ± 24.48	54.73 ± 23.20	51.54 ± 23.16
GM FF (%)	51.86 ± 24.81	53.26 ± 23.25	59.32 ± 19.90	57.11 ± 20.33
Lateral compartment				
Per FF (%)	44.39 ± 27.15	45.87 ± 25.18	49.15 ± 24.85	47.4 ± 23.34

Abbreviations: AM = adductor magnus; BF = biceps femoris; CK = creatine phosphokinase; ED = extensor digitorum muscles; FF = fat fraction; GM = gastrocnemius medialis; GL = gastrocnemius lateralis; Gra = gracillis; NA = nonapplicable; NSAD = North Star Assessment for Dysferlinopathy score; Per = peroneuos; Sar = sartorius; SM = semimembranosus; Sol = soleus; ST = semitendinosus; TA = tibialis anterior; TP = tibialis posterior; U/L = units per liter; VI = vastus intermedius; VL = vastus lateralis; VM = vastus medialis; yo = years old.

### Statistical Analysis and Model Building

Statistical analysis was conducted using IBM SPSS Statistics software version 29. Figures were built using Minitab statistical software version 21.2 and R studio version 2024.09.1 + 394.

We analyzed whether there was any statistically significant difference in FF for each muscle bilaterally through the paired samples *t* test, with significance level set at *p* < 0.05. We also generated descriptive statistics of the demographic characteristics of our cohort. Continuous variables were described using mean (x̄) and SD, and qualitative variables were described as percentages (%).

FF is expressed as a percentage; therefore, its values are naturally bounded between 0% and 100%. This poses a challenge to statistical methods that assume a normal distribution of the data, as percentages cannot extend beyond these limits. The logit transformation overcomes this hurdle by transforming FF into a new variable (Y) with an unbounded range using the formula Y = log((FF/100–FF)). In this way, extreme FF values are more evenly spread allowing the use of standard statistical methods that assume a normal distribution.^22^ After modeling the Y variable, we applied the inverse logistic transformation and predicted the FF values across all time points in the original percentage scale.

First, we built a simple linear-mixed model (LMM) for each individual muscle and each global muscle segment (thigh and lower leg) including Y values (logit transformation of FF) as the dependent variable and the age of the patient at the MRI scan as the independent variable. Each patient's data included multiple visits (Level 1), meaning that visits were nested within patients (Level 2). A random intercept and slope were included to account for between-participants variability at baseline and over time, respectively.

Later, we performed additional LMM analyses, adding other variables to the model. Based on previously published research^7,9-13^ on disease progression in dysferlinopathy and skeletal muscle MRI analysis, in addition, to age at MRI scan, the following candidate variables were considered for inclusion: sex, ethnicity (White, Asian, Black, or Hispanic), residual dysferlin protein expression measured using immunoblotting (first we assessed muscle biopsy results and, if not available, peripheral blood monocytes), predicted phenotype based on genetic variants (if a patient had 2 nonsense variants, 2 missense variants on C2A or C2B domain^23,24^ or one of each, patient was classified as severe, whereas if the patient had any 2 missense variants other than the previously described or 1 missense and 1 nonsense, patient was classified as mild), intensity of physical activity as a teenager (patients were classified as 0: no physical activity, 1: vigorous activity once a month or moderate once a week, 2: moderate activity multiple times per week or vigorous once a week, 3: vigorous activity multiple timer per week^11^), age at symptom onset, disease duration, NSAD score, and velocity in performing 6-minute walking test (6MWT). We included them one at a time, and after each model was built, we used the χ² likelihood ratio test to determine whether the new model was a better fit to the data than the previous one, and if it was, we added another variable and repeated the process. On the other hand, if the fit was not improved by the last added variable, we then would remove it and continue with the next. Once we had added all the variables that proved to be relevant, we started removing those with a fixed effects value above 0.05 and, if the fit of the model did not worsen, we completely removed them from the final LMM. Finally, we built 2 types of multivariate models: one that included only demographic and baseline clinical variables and another one also including clinical variables reflecting changes in different parameters over a 1-year period, with the final aim of improving the model's fit to the data. Because longitudinal analysis of global muscle segments' (thigh and lower leg) FF has shown to be sensitive to change for dysferlinopathy (compared with an individual muscle approach),^25^ we decided to perform this multivariate analysis solely in these 2 segments. To quantify the model's performance by analyzing the residual values in the original percentage scale, we applied the inverse logit transformation to the predicted values and compared them with the observed FF values across all patients and time points, calculating the mean absolute error and the SD.

## Results

In this study, 109 patients were included. Fifty-seven were female, and on average, each patient had 3.27 MRI scans done over a 3-year period in 1 of 7 participating centers (eTable 2). A summary of the cohort's demographics and mean right-to-left FF values for each muscle and segment at each visit can be found in Table 1. A little over 3 quarters (77.8%) of patients were ambulant at baseline, and 1 patient only used noninvasive ventilation.

The paired samples *t* test showed no difference in muscle replacement by fat for each of the assessed muscles between right and left at baseline (*p* > 0.05). Therefore, we decided to calculate the mean FF value for each muscle using the right and left data when available and use those values for the analysis.

**Figure 1:**
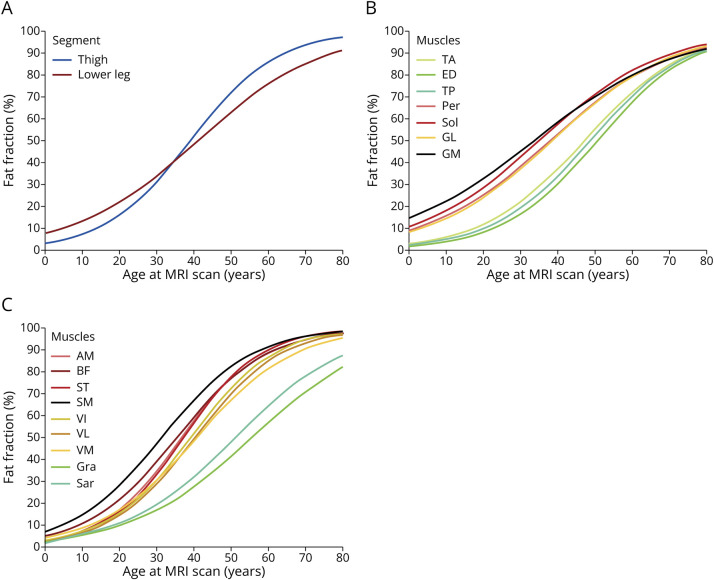
Average Trajectories (Fixed-Effects LMM) for Thigh and Lower Leg as 2 Segments (A), Lower Leg Muscles (B), and Thigh Muscles (C) AM = adductor magnus; BF = biceps femoris; ED = extensor digitorum; GM = gastrocnemius medialis; GL = gastrocnemius lateralis; Gra = gracillis; LMM = linear mixed model; Per = peroneals; Sar = sartorius; SM = semimembranosus; Sol = soleus; ST = semitendinosus; TA = tibialis anterior; TP = tibialis posterior; VI = vastus intermedius; VL = vastus lateralis; VM = vastus medialis; yo = years old.

Figure 1A shows the average trajectory of the thigh and lower leg while Figure 1, B and C show the average trajectory of each muscle (B, lower leg and C, thigh). eFigure 1 shows the average trajectory on top of the patients' observed FF values for each individual muscle.

In teenage years and early adulthood, lower leg muscles show higher levels of muscle replacement by fat than thigh muscles; however, the rate of disease progression for thigh muscles is steeper; therefore, after the third decade of life, thigh muscles progressively become more involved than lower leg muscles (Figure 1A).

Two distinct muscle groups arise after analyzing FF trajectory in the lower legs (Figure 1B), the anterior compartment muscles plus the TP, responsible for plantar dorsiflexion, extension of the toes and foot inversion, and the posterior and medial compartment muscles, responsible for plantar flexion and foot eversion, respectively, which show more severe muscle replacement by fat. Something similar can be seen in the thigh muscles (Figure 1C), as the sartorius and gracilis muscles have a different trajectory showing lower FF values as the disease progresses compared with the other thigh muscles, resembling the trajectory of the lower leg anterior compartment. The femoral quadriceps muscles, responsible for knee extension, were less involved than the hamstring and AM muscles, responsible for knee flexion and adduction, respectively. To complement Figure 1, Table 2 provides the corresponding quantitative data, including the mean annual rate of progression and the expected FF value at 40 years of age for each muscle, as described by our models (eTable 3). Table 2 serves as a reference for understanding and comparing the progression rates and trajectories between muscles. In summary, muscles can be grouped into 3 categories: (1) spared muscles: the anterior lower leg muscles, the TP, the gracilis, and the sartorius; (2) severely involved muscles in early disease stages: the posterior and medial lower leg muscles; and (3) muscles increasingly involved with disease progression: the thigh muscles (except for the gracilis and sartorius). This group can be further divided into 2 subgroups based on their trajectories: the anterior compartment and the posterior compartment plus the AM. Overall, muscles that participate in the same joint movement seem to progress similarly over time.

**Table 2:** Mean Annual FF Progression Rate per Muscle and Predicted Mean Value of FF at 40 Years Old

Segment		
Muscle	Mean annual FF progression rate (%)	FF value at 40 yo according to the model (%)
Thigh	1.17	51.7
Anterior compartment		
VM	1.14	48.8
VL	1.18	49.3
VI	1.18	51.9
Posterior compartment		
BF	1.15	59.0
SM	1.14	67.0
ST	1.20	56.6
Medial compartment		
AM	1.19	57.7
Sar	1.05	32.1
Gra	0.99	27.4
Lower leg	1.04	48.2
Anterior compartment		
ED	1.11	30.3
TA	1.11	37.4
Posterior compartment		
TP	1.11	34.1
GL	1.06	52.3
Sol	1.03	57.5
GM	0.96	58.1
Lateral compartment		
Per	1.04	53.1

Abbreviations: AM = adductor magnus; BF = biceps femoris; ED = extensor digitorum muscles; FF = fat fraction; GM = gastrocnemius medialis; GL = gastrocnemius lateralis; Gra = gracilis; Per = peroneals; Sar = sartorius; SM = semimembranosus; Sol = soleus; ST = semitendinosus; TA = tibialis anterior; TP = tibialis posterior; VI = vastus intermedius; VL = vastus lateralis; VM = vastus medialis; yo = years old.

**Figure 2:**
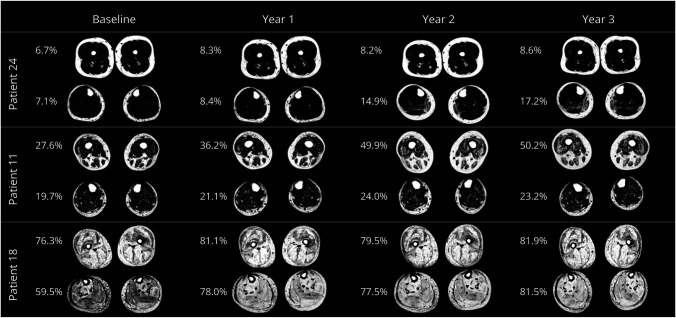
Fat Fraction Maps Obtained Yearly for 3 Patients With Different Degrees of Fat Replacement The corresponding fat fraction values are shown to the left of each segment.

We plotted each patient's modeled trajectory against the observed data obtained in the study for thigh (eFigure 2A) and lower leg muscles (eFigure 2B). Using the average FF trajectory for each muscle, we created an animated image of the thigh and lower leg showing the mean progression of muscle replacement by fat as patient's age (eFigure 3). Moreover, Figure 2 shows the change in the FF maps reconstructed from the Dixon images for 3 patients with different severities of muscle replacement by fat.

Tables 3 and 4 summarize the characteristics of the 3 built models for thigh and lower leg (simple, including baseline variables and including longitudinal variables). Moreover, eTable 4 shows intermediate results in the building of each model and how the addition or removal of variables affected the χ² likelihood ratio test. The thigh baseline model incorporated age at MRI scan, age at symptom onset, baseline NSAD score, and baseline 6MWT velocity, whereas the longitudinal thigh model included age at MRI scan, age at symptom onset, baseline NSAD score, and changes over the first year in 6MWT velocity. The lower leg baseline model included age at MRI scan, age at symptom onset, sex, teen exercise intensity, baseline NSAD score, and baseline 6MWT velocity and the longitudinal model included age at MRI scan, age at symptom onset, sex, teen exercise intensity, baseline NSAD score, and changes over the first year in 6MWT velocity. According to the model, women had on average, an absolute value of FF 8.8% higher than men in the lower legs, with a maximum difference of 12.4% at age 45 years. Neither the addition of ethnicity, residual dysferlin expression, or predicted phenotype based on the genetic variants improved the models' fit to the data.

**Table 3:** Estimates of Fixed Effects of the Bivariate and Multivariate Models for Thigh and Lower Leg

Parameter	Estimate	Std. error	95% CI	*p* Value
A. Thigh simple model				
Intercept	−3.34	0.274	−3.9 to −2.80	<0.001
Age at MRI scan	0.08	0.007	0.07–0.10	<0.001
B. Thigh baseline model				
Intercept	−1.16	0.248	−1.65 to −0.67	<0.001
Age at MRI scan	0.08	0.007	0.06–0.09	<0.001
Age at symptom onset	−0.06	0.010	−0.08 to −0.03	<0.001
NSAD bl	−0.03	0.009	−0.05 to −0.01	<0.001
Vel 6MWT bl	−0.07	0.289	−0.64 to 0.51	0.814
C. Thigh longitudinal model				
Intercept	−1.28	0.251	−1.77 to −0.78	<0.001
Age at MRI scan	0.08	0.007	0.07–0.09	<0.001
Age at symptom onset	−0.06	0.011	−0.08 to −0.04	<0.001
NSAD bl	−0.03	0.005	−0.04 to −0.02	<0.001
Δbl-y1 vel 6MWT	−1.34	0.685	−2.70 to 0.02	0.053
D. Lower leg simple model				
Intercept	−2.56	0.277	−3.10 to −2.01	<0.001
Age at MRI scan	0.06	0.007	0.04–0.07	<0.001
E. Lower leg baseline model				
Intercept	−0.56	0.313	−1.18 to −0.06	0.076
Age at MRI scan	0.05	0.008	0.03–0.06	<0.001
Age at symptom onset	−0.03	0.011	−0.06 to −0.01	0.003
Male sex	−0.48	0.133	−0.74 to −0.21	<0.001
Teen exercise level	−0.04	0.060	−0.16 to 0.08	0.491
NSAD bl	−0.02	0.009	−0.04 to −0.01	0.009
Vel 6MWT bl	−0.13	0.285	−0.70 to 0.44	0.648
F. Lower leg longitudinal model				
Intercept	−0.71	0.326	−1.35 to −0.07	0.032
Age at MRI scan	0.06	0.008	0.04–0.07	<0.001
Age at symptom onset	−0.04	0.012	−0.07 to −0.02	<0.001
Male sex	−0.55	0.140	−0.83 to −0.27	<0.001
Teen exercise level	−0.04	0.065	−0.17–0.09	0.478
NSAD bl	−0.02	0.005	−0.03 to −0.01	<0.001
Δbl-y1 vel 6MWT	−1.11	0.673	−2.45 to 0.23	0.101

Abbreviations: 6MWT = 6-min walk test; bl: baseline; Δ = delta; NSAD = North Star Assessment for Dysferlinopathy score; Std = standard; vel = velocity; y1 = year 1.

**Table 4:** Summary of χ² Likelihood Ratio Test and Performance Analysis of Both Segments

Model	Parameters	−2 Log L	X^2^	df	*p* Value	MAE, (%)	SD, (%)
A. Thigh models							
Simple	1	290.7	—	—	—	2.03	2.94
Baseline	4	176.4	114.3	3	<0.001	2.18	3.11
Longitudinal	4	160.0	16.4	0	<0.001	2.16	3.08
B. Lower leg models							
Simple	1	414.0	—	—	—	2.66	3.86
Baseline	6	293.6	120.4	5	<0.001	2.71	3.93
Longitudinal	6	278.1	15.5	0	<0.001	2.70	3.93

Abbreviations: df = degrees of freedom; Log L = logarithm of the likelihood function; MAE = mean average error.

In both tables, the X^2^, df, and *p* values are comparing simple vs baseline (results in baseline row) and baseline vs longitudinal (results in longitudinal row).

## Discussion

We have analyzed longitudinal thigh and lower leg Dixon-based FF data from patients with LGMDR2 acquired annually over a 3-year period. We have modeled individual muscles' trajectories over time and created multivariate models for the thigh and lower leg, assessing how different variables influence FF progression. These results allowed us to make the following conclusions: (1) Muscles that participate in generating the same joint movement have similar FF trajectories as the disease progresses, suggesting that there could be biomechanical characteristics determining FF progression in dysferlinopathy; (2) female sex is linked to higher FF values in the lower legs but not in the thighs; (3) the younger the age at symptom onset, the higher the predicted FF values; and (4) the baseline NSAD score strongly contributes to explaining FF values over time.

Our findings suggest that muscles involved in producing the same movements progress with a similar trajectory, probably because of their exposure to comparable mechanical loads and tension over time. Given that dysferlin plays a critical role in repairing muscle membrane damage, the failure of this repair mechanism in individuals with dysferlinopathy may lead to synchronized degeneration patterns.^26,27^ This supports the idea that the cumulative mechanical stress these muscles endure throughout life might contribute to the observed trajectory of muscle degeneration.

Modeling of lower leg FF values differed between male and female participants while this was not the case for the thigh model. There were no statistically significant differences in age at assessment, age at symptom onset, or disease duration between men and women included in this study in the whole cohort. The available data on differences between male and female patients with dysferlinopathy are scarce and controversial. On the one hand, semiquantitative MRI-based analysis showed that female patients had more severe muscle replacement by fat in some muscles of the thigh and lower leg than their male counterparts.^14^ On the other hand, women were found to perform better in muscle function tests (NSAD) while showing similar rates of progression as men.^10^ This discrepancy could be partly explained by the fact that the NSAD score better reflects the function of proximal rather than distal lower limb muscles. To our knowledge, there is no published data on molecular or genetic differences between sexes on the skeletal muscle structure of patients with dysferlinopathy. Nevertheless, differences between sexes and muscle lipid accumulation have been described in Bla/J dysferlin-deficient mice, with females showing higher lipid content and upregulation of genes linked to lipid metabolism, especially in the quadriceps and gastrocnemius muscles.^28^ Moreover, it has been suggested that fast type II fibers are more prone to muscle damage than slow type I fibers in muscular dystrophies^29^; however, in LGMDR2 mice models and humans, type I myofibers exhibit a higher content of lipid droplets.^30,31^ Although skeletal muscle fiber type composition is similar between men and women, it has been described that the ratio of type II/I fibers is greater in men than in women.^32^ How meaningful these differences are and whether they might play a role in the progression of dysferlinopathy is currently unknown, highlighting the need for further research on biological modifiers that can influence disease progression and response to therapies.

Intense exercise during teenage years in people with LGMDR2 has been linked to earlier symptom onset, and part-time as well as full-time wheelchair use.^11,12^ In the multivariate models, teenage exercise levels had a fixed effects value above statistical significance for both thigh and lower leg, but its removal from the models worsened the fit only to the lower leg data. A previously published work^12^ looking specifically at the effect teenage exercise could have on muscle replacement by fat measured through T_1_-weighted images using Mercuri score concluded only some pelvic muscles showed significantly higher fat content in the higher exercise levels group and no differences were seen between groups in the thigh or lower leg muscles. We agree with their hypothesis in that this lack of difference could be because of the thigh and lower leg muscles having an earlier involvement than the pelvic ones in the natural history of the disease and so, as patients in this cohort have been symptomatic for 16 years on average, there could be an initial difference in earlier stages that we were unable to capture.

Age at symptom onset had a negative effect on FF trajectory; therefore, the older the age at symptom onset, the lower the predicted FF value at a given time point, and this variable remained statistically significant in the 4 multivariate models, highlighting its weight in influencing FF. Numerous studies have shown that longer disease duration is associated with greater disease severity.^7,10,14,33^ However, the relation between age at symptom onset and disease duration has proven to be more complex, with fewer evidence supporting a more severe phenotype^8,10^ and faster progression^10^ in patients with an earlier onset of symptoms. Our findings support the potential importance of age at symptom onset, along with disease duration, in shaping disease trajectory, and therefore, both parameters should be considered in the analysis of patients with LGMDR2. The reason why some patients present with symptoms earlier than others still remains an unanswered question, but we think that this is due to the interaction of multiple factors rather than solely the underlying pathogenic variant, a hypothesis that is also supported by our results because the inclusion of genetic data in our models did not reveal any clinically significant differences between groups.

Baseline NSAD score had a negative effect on FF modeling. Those patients with lower baseline NSAD scores showed higher FF values and were therefore more severely affected. The baseline NSAD score remained statistically significant in all models even after including longitudinal variables, suggesting that it has a strong and persistent power in explaining FF values. This finding is consistent with previous studies reporting a strong correlation between clinical severity and MRI-based measures in LGMDR2.^15^

The inclusion of clinical variables in the multivariate models provided valuable insight into the interactions between these factors and FF values. Furthermore, the χ² likelihood ratio test indicated a significant improvement in model fit when moving from bivariate to multivariate models, highlighting the contribution of additional variables to the overall explanatory power of the model. By contrast, despite this improvement in overall data fit, the measures of dispersion did not improve, meaning that the average residual values (i.e., the difference between the observed FF value and the 1 predicted by the model for every patient at each time point) were not overall reduced after including additional variables. This suggests that, although the new variables improved the fit of the models at the group level, they were not able to improve capturing individual level variability in FF. A larger sample size, more longitudinal data points per patient, and probably more complex modeling approaches like nonlinear or machine learning based could potentially clarify this limitation. That said, we are certain this is not purely related to the design of the trial and statistical analysis because we think other variables, such as health comorbidities, medication, not only intensity but also type of exercise carried out as teenagers and in adult life, nutrition and diet, hormonal, and other genetic and epigenetic variants impacting muscle and metabolism, are also influencing disease severity and progression.

Although these models reflect that most patients will show an increase in skeletal muscle fat replacement as they age, the rate at which this is expected to happen varies considerably between patients (eFigure 2). Some patients' modeled trajectories have a negative slope, meaning that according to the model their muscle pathology should improve over time. This is mainly seen in patients who are relatively stable in the progression, usually because they are either very severely or mildly affected, and small fluctuations in FF measurements that reflect a supposed decrease in fat content lead the model into interpreting that the patient is improving. This limitation could be overcome by following patients for longer periods of time and smoothing these yearly fluctuations in the overall trajectory.

Other limitations we faced in this study were those related to the multicenter nature of COS 1. There is variability inherent to the fact that different MRI systems were used at different sites (eTable 1). The same principle is applicable to clinical outcome measures because although all health care professionals involved were trained in how to assess patients correctly and objectively, interobserver variability is a well-recognized bias and one of the most frequent problems large scale studies like COS face. Moreover, although COS is the largest natural history study in dysferlinopathy, a 3-year follow-up is still a short period of time in the natural history of this non–life-limiting rare disease, only offering a snapshot of a long and complex disease progression. The youngest included patient was in its late teens; therefore, the absence of FF data from younger patients means that modeling of FF in patients younger than 15 years was solely based on what we would expect following the muscle fat replacement process in adult life, which could be biased. Finally, this model was built using a single cohort, and its predictive accuracy has not yet been evaluated using independent data. Future validation with an external cohort will be essential to assess generalizability and reliability in capturing FF progression among a broader population. After the completion of COS 1, the Jain Foundation set up COS 2 (NCT01676077), another natural history study with the aim of gaining further understanding of some aspects where COS 1 has fallen short of, focusing on developing standards of care, getting closer to clinical trial readiness, and validating the developed outcome measures from COS 1. This would be an interesting cohort to validate these results with.

In conclusion, this study offers models to better understand and describe progression of muscle FF of the thighs and lower legs of patients with dysferlinopathy using clinical and radiologic data. The analysis of multivariate models provided relevant information on the way different factors shape muscle degeneration. The results presented here contribute to the body of information that can be used for the design of clinical trials and the analysis of results. Based on these data, we suggest that, in the design of future placebo-controlled clinical trials, not only the patient's age is considered for randomization but also age at symptom onset, sex, and baseline NSAD score, meaning the ideal pair of patients would be 2 people of the same sex and age who started showing symptoms at a similar moment in life and have similar disease severity. Moreover, although the effect of physical activity early on in life on posterior disease progression is still not completely understood, some evidence points toward an association between it and faster decline and disease severity and should therefore be assessed during screening and, ideally, included in the randomization process, or at least controlled for during final data analysis.

## Appendix Coinvestigators

**Table:**

Coinvestigators are listed at Neurology.org/NG.

## Glossary

AM: adductor magnus
BRC: biomedical research centre
COS 1: clinical outcome study 1
FF: fat fraction
LGMDR2: limb-girdle muscular dystrophy R2
LMM: linear-mixed model
MAE: mean absolute error
NIHR: National Institute for Health and Care Research
NSAD: North Star Assessment for Dysferlinopathy
TP: tibialis posterior
